# Enhanced Gamma Oscillatory Activity in Rats with Chronic Inflammatory Pain

**DOI:** 10.3389/fnins.2016.00489

**Published:** 2016-11-01

**Authors:** Jing Wang, Jing Wang, Guo-Gang Xing, Xiaoli Li, You Wan

**Affiliations:** ^1^Department of Neurobiology, School of Basic Medical Sciences/Beijing Institute for Brain Disorders, Capital Medical UniversityBeijing, China; ^2^Neuroscience Research Institute, Peking UniversityBeijing, China; ^3^Peking University Sixth Hospital/National Clinical Research Center for Mental Disorders, Key Laboratory of Mental Health, Ministry of Health (Peking University)Beijing, China; ^4^State Key Laboratory of Cognitive Neuroscience and Learning & IDG/McGovern Institute for Brain Research, Beijing Normal UniversityBeijing, China; ^5^Key Laboratory for Neuroscience, Ministry of Education/National Health and Family Planning Commission, Peking UniversityBeijing, China

**Keywords:** gamma activity, chronic pain, inflammatory pain, cross-frequency coupling, rat, hyperalgesia, spontaneous electrocorticogram

## Abstract

It has been reported that oscillatory gamma activity participates in brief acute pain and tonic ongoing pain. It is of great interest to determine whether the gamma activity is involved in chronic pain since chronic pain is a more severe pathological condition characterized by pain persistency. To investigate the oscillatory gamma activity in chronic pain, in the present study, we recorded spontaneous electrocorticogram (ECoG) signals during chronic pain development in rats with chronic inflammatory pain induced by monoarthritis. Power spectrum analysis of ECoG data showed that gamma power increased significantly at the late stage of chronic inflammatory pain. The increased gamma activity occurred mainly at electrodes over primary somatosensory cortices. In rats with chronic pain, the gamma power was positively correlated with the hyperalgesia measured by laser energy that elicited hindpaw withdrawal response. Furthermore, an increased coupling between the amplitude of gamma power and the phase of theta oscillations was observed in chronic inflammatory pain condition. These results indicate an enhanced spontaneous gamma activity in chronic pain and suggest a potential biomarker for the severity of chronic pain.

## Introduction

Oscillatory brain activities in gamma frequency band play an important role in selecting and integrating sensory-relevant information into a coherent perception (Herrmann et al., 2004). It has been reported that gamma band activation participates in pain perception. The power of gamma oscillations increases after brief nociceptive laser stimulations in healthy human beings (Gross et al., 2007; Tiemann et al., 2010; Schulz et al., 2011; Zhang et al., 2012; Hu et al., 2014; Liu et al., 2015a,b) and in normal rats (Wang et al., 2011), indicating that gamma oscillations are related to experimental acute pain lasting for milliseconds to seconds. Such gamma activity is focused on somatosensory cortex and positively correlated with the perceived pain intensity measured by the subjective rating of pain sensation on human beings (Gross et al., 2007; Zhang et al., 2012; Hu et al., 2014; Liu et al., 2015a) and with convert pain processing in patients with chronic disorders of consciousness (Naro et al., 2016). Recent recordings from deep brain structures on human beings discovered that laser nociceptive stimulation on skin could induce gamma activation in brain regions like the parasylvian, the thalamus, right amygdala, and the hippocampus (Liu et al., 2015a,b).

Besides brief acute pain, gamma activity also participates in tonic ongoing pain which lasts for minutes. Recent studies found that human subjects had strong gamma activity when they received heat stimulation for 5 or 10 min (Peng et al., 2014; Schulz et al., 2015) and when they were under medium tonic muscle pain condition (Li et al., 2016). These gamma activity engaged the prefrontal cortex and the frontal-central areas, which are responsible for the emotional and cognitive components of pain, were positively correlated with subjective pain intensity (Peng et al., 2014; Schulz et al., 2015; Li et al., 2016). Thus, gamma activity is considered to participate in experimental acute pain and tonic pain as well.

Gamma oscillation was shown to participate in the abnormal working memory of chronic pain (Cardoso-Cruz et al., 2013); however, whether oscillatory gamma oscillations play a role in the pain perception in chronic pain is unknown and of great interest. Unlike acute and tonic pain, chronic pain is a pathological, ongoing, and long-lasting condition (Merskey and Bogduk, 1994), featured by its persistence and maintenance of pain. Chronic pain is a complex disease with enhanced and prolonged processing of pain-related sensory, emotion, and cognitive components (Apkarian et al., 2011). Considering gamma activity participates in the sensory, emotional, and cognitive components of pain processing (Gross et al., 2007; Liu et al., 2015a; Schulz et al., 2015), we hypothesize that gamma activity is also involved in chronic pain.

In the present study, we aimed to investigate oscillations at the gamma frequency band and its modulation in the development of chronic pain condition. Considering electrocorticogram (ECoG) signals from rats could provide high level of signal to noise and facilitate longitudinal study, we measured ECoG signals from rats with chronic inflammatory pain development, analyzed the oscillatory gamma activities, and correlated it with the hyperalgesia of chronic pain rats.

## Materials and methods

### Animals

Thirteen adult male Sprague-Dawley rats (300–350 g) were provided by Department of Experimental Animal Sciences, Peking University Health Science Center. Animals were housed in individual cages (12-h light/12-h dark, room temperature around 22°C) with free access to food throughout the experiment. All experimental procedures were approved by the Animal Care and Use Committee of Peking University Health Science Center and were in accordance with the Guidelines of International Association for the Study of Pain.

### Surgery for recording electrode implantation

**Figure 1:**
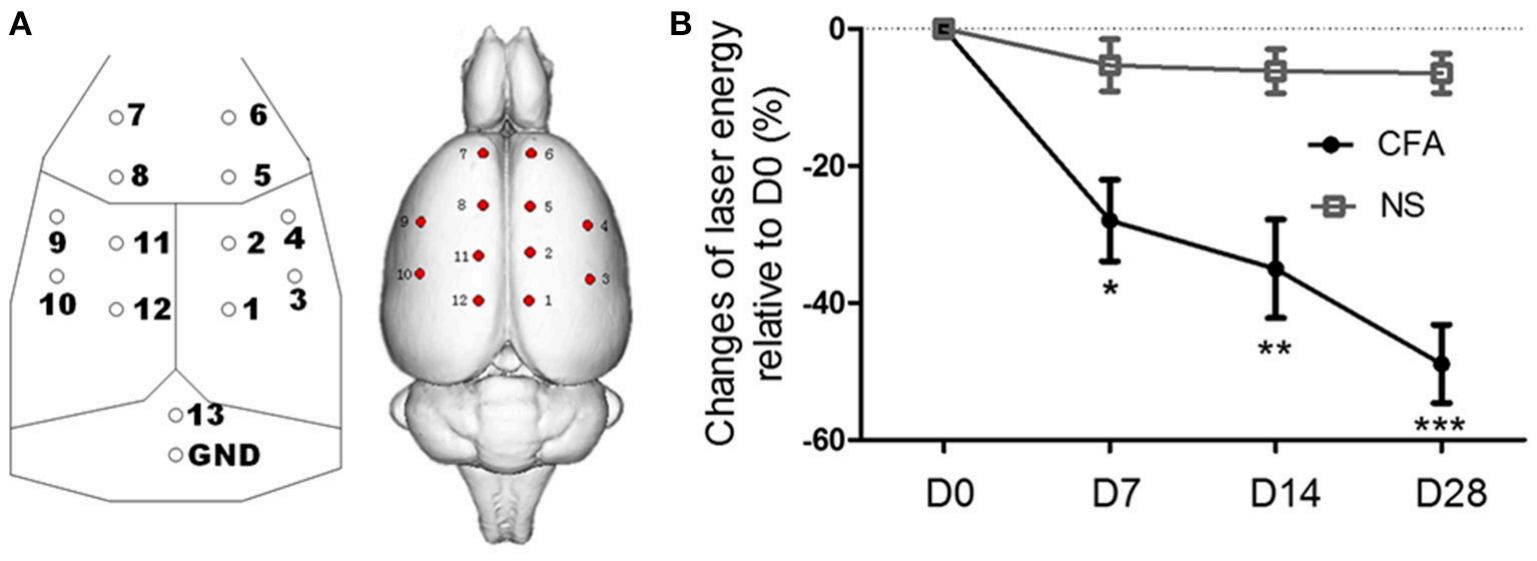
**EEG electrode locations and the thermal hyperalgesia in chronic inflammatory pain rats. (A)** Sketch map of EEG electrode locations: electrodes 4 and 9 [anterior (A) 0.0 mm, lateral (L) ±4.5 mm]; electrodes 3 and 10 (A −3.0 mm, L ±4.5 mm); electrodes 6 and 7 (A +4.5 mm, L ±1.5 mm); electrodes 2, 5, 8, 11 (A ±1.5 mm, L ±1.5 mm); electrodes 1 and 12 (A −4.5 mm, L ±1.5 mm). Electrode 13 was set as a reference electrode and positioned 2 mm caudal to the lambda. The ground electrode (GND) was positioned 4 mm caudal to the lambda. **(B)** The laser energy that elicited pain response in CFA rats was significantly lower than that in control rats from day 7 to day 28, indicating thermal hyperalgesia developed. ^*^p < 0.05; ^**^p < 0.01; ^***^p < 0.001 compared with the NS control group.

After rats were anesthetized with sodium pentobarbital (50 mg/kg, i.p.), 14 electrodes made of stainless steel (tip diameter 1 mm, impedance 300–350 Ω) were implanted epidurally. The locations of the electrodes were determined according to the method by Shaw et al. (1999) and Hu et al. (2015) (Figure 1A). Electrodes were then fixed to the skull with dental cement. The surgery lasted for about 35 min and penicillin was injected (60,000 U, i.m.) to prevent possible infections.

In the following 3 days after surgery, rats were housed individually and allowed to habituate to the recording environment for 1 h every day.

### Chronic inflammatory pain model induced by monoarthritis

Chronic inflammatory pain model of monoarthritis was established by injection of complete Freund's adjuvant (CFA) (Butler et al., 1992). In brief, the procedure was as follows: mycobacterium butyricum (60.0 mg), paraffin oil (6.0 ml, Sigma-Aldrich), NaCl 0.9% (4 ml), and Tween 80 (1 ml) were mixed and then autoclaved. After the rat was anesthetized with sodium pentobarbital (50 mg/kg, i.p.), the CFA mixture (50 μl) was injected into left tibio-tarsal joint cavity to establish monoarthritis chronic inflammatory pain model (CFA group). Rats in the control group were injected with an equal volume of 0.9 % NaCl solution instead (NS group).

### Measurement of pain threshold and hyperalgesia

We applied the nociceptive laser withdrawal test to evaluate the pain threshold and hyperalgesia of rats (Kao and Jaw, 2011; Wang et al., 2011). It estimates the intensity of pain based on the intensity of nociceptive stimuli which could induce the hindpaw withdrawal response. Prior to EEG recording, thermal pain threshold of rat was determined by an ascending series of laser beams (wavelength 10.6 μm, beam diameter 2.5 mm, pulse width 20 ms). The stimulation interval was at least 40 s to avoid possible thermal sensitization. The energy level that generated 4–5 hindpaw withdrawal responses out of six stimuli was chosen. The higher energy level that elicited pain response indicated the lower hyperalgesia of a rat. Each stimulus was targeted at a slightly different position of the planta to avoid unnecessary injury or sensitization of the skin.

### Recording of spontaneous ECoG

Seven days after implantation of the electrodes, ECoG was recorded at four time points, including before CFA or NS injection as well as 7, 14, and 28 days after injection (denoted as D0, D7, D14, and D28, respectively). The electrical signals from 13 electrodes were recorded through digital pre-amplifier using EEG/ERP software (CogniTrace ERP, ANT Inc., The Netherlands). The rats were awake and walked freely in a plastic cage (40 × 40 × 30 cm^3^ in volume) with an apertured bottom (5 mm) without any stimulation during the recording. Prior to the spontaneous ECoG recordings, rats were allowed to habituate the environment for about 5 min. Then, ECoG was recorded for another 20 min. All signals were referenced to the electrode located 2 mm caudal to the lambda. Data were recorded at a sampling frequency of 512 Hz. The behavior of the rat was video-taped simultaneously.

### Pre-processing of the EEG data

Pre-processing of the EEG data was performed using the MatLab-based EEGLAB toolbox (http://sccn.ucsd.edu/eeglab/). Only when a rat was awake and quiet on video display (detected by a blinded experimenter), the spontaneous EEG data during this period was picked out and band-pass filter was set between 1.5 and 45 Hz to remove the 50 Hz line noise artifacts. After removing environmental artifacts of large amplitude, the EEG signals were re-referenced to the averaged signal across all channels. The length of final data segments was not significantly different between groups [*F*_(1, 44)_ = 0.73, *p* > 0.05] and among different time points [*F*_(3, 44)_ = 1.07, *p* > 0.05].

### Wavelet power spectrum for spontaneous EEG

A continuous wavelet transform was used to decompose the EEG data. In this approach, the wavelet coefficients were produced through the convolution of a parent wavelet function with the analyzed signal. Here, a Morlet wavelet was applied with a wavelet central angle frequency of 8 Hz as in our previous report (Li et al., 2007). Based on the Morlet wavelet transform, wavelet power of the EEG data at different frequencies was obtained and used to describe the variance of the data. The wavelet power spectrum was used to obtain the power of oscillatory activity at gamma frequency band (30–45 Hz) in the spontaneous EEG data. The frequency resolution was 0.5 Hz. Frequency band was defined as delta (1.5–4 Hz), theta (4–8 Hz), alpha (8–12 Hz), beta 1 (12–20 Hz), beta 2 (20–30 Hz), and gamma (30–45 Hz).

### Phase amplitude coupling

Phase amplitude coupling was estimated by the method described by Canolty et al. (2006), because this method can not only detect the presence of phase amplitude coupling, but also assess the intensity of the coupling (Tort et al., 2010). Continuous 1-min EEG epoch was chosen for the analysis. EEG signal was firstly filtered at frequencies from 2 to 20 Hz and from 30 to 45 Hz with step of 1 Hz. Then, both the amplitude and the phase of the low frequency and high frequency were extracted by Hilbert transformation. A complex-valued signal was further constructed by combining the high frequency amplitude time series with the low frequency phase time series. The modulation index was obtained by measuring the degree of asymmetry of the probability density function of the complex-valued signal. Comparing the modulation index of signal with that of 100 surrogated data generated by shifting the amplitude time series, the normalized modulation index was obtained.

### Statistical analysis

We tested the assumptions of analysis of variance (ANOVA) for all the variables, including Levene's test for testing the homogeneity of variance, the Shapiro-Wilk for testing the normality and the Mauchly's for testing sphericity. Since all variables met the normal distribution and sphericity which is the key variation assumptions for repeated ANOVA, statistical analysis was performed using two-way mixed ANOVA with recording time (day) as a repeated factor. *Post hoc* tests were performed using Bonferroni correction. *p* < 0.05 was considered as statistically significant. For cross-frequency coupling, two-way ANOVA was performed with channels and groups as variables.

## Results

### Enhanced gamma activity in chronic inflammatory pain rats

First, in order to test CFA rats developed hyperalgesia of chronic pain, pain intensity were compared between CFA rats and control rat. As shown in Figure 1B, pain intensity, the laser energy that elicited pain response, was significantly different between CFA rats and control rats [*F*_(1, 11)_ = 18.25, *p* < 0.01], and there was significant interaction between groups and days [*F*_(3, 33)_ = 9.65, *p* < 0.001]. The laser energy of CFA rats was significantly lower than that of control rats from day 7 (*p* < 0.05, Bonferroni *post hoc* test) to day 28 (*p* < 0.001, Bonferroni *post hoc* test), indicating a hyperalgesia in chronic pain rats.

**Figure 2:**
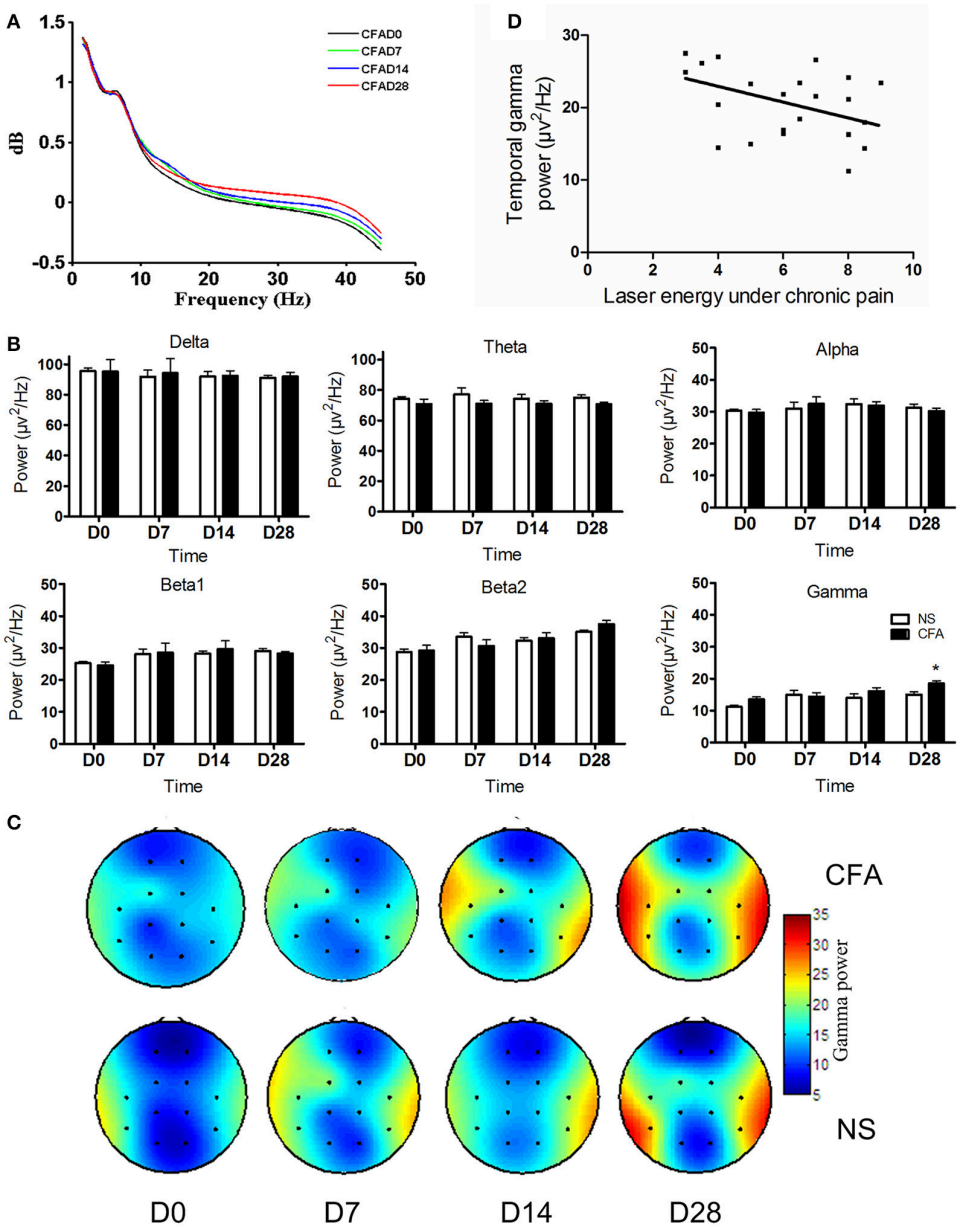
**Enhanced gamma power in chronic pain. (A)** Power spectrum during the development of chronic pain. **(B)** Grand average power (mean ± SEM) of each frequency band in CFA group and in NS control group at different days after CFA injection. Gamma band power was significantly higher at day 28 in the CFA chronic pain group compared with that in NS control group (^*^p < 0.05). **(C)** Gamma power over channels 3 and 10 was increased in the CFA chronic pain group (upper) compared with that in the NS group (below). **(D)** Negative correlation between gamma power over channels 3 and 10 and the hyperalgesia in chronic pain rats at D7, D14, and D28 after CFA injection (Pearson γ = −0.45, *p* < 0.05).

Next, we investigated whether the oscillatory activity changed dynamically during the development of chronic pain. Thus, the power spectrum of spontaneous ECoG at each frequency was calculated for CFA rats at each time point. The averaged power spectrum across all ECoG recording channels showed that power at higher frequency became stronger during the development of chronic pain (Figure 2A). In order to explore which frequency band altered under chronic pain condition, the power at each frequency band was compared between two groups. Due to the spontaneous ECoG signal, we failed to apply band separation method (Magri et al., 2012) but applied the traditional frequency band-wise analysis alternatively. So, as shown in Figure 2B, it was found that gamma band activities in CFA rats were significantly higher, but activities at other frequency bands were not different between two groups. The power at gamma frequency was significantly higher in the CFA group than that in the NS group [*F*_(1, 11)_ = 4.90, *p* < 0.05], and the main effect of time showed that gamma power increased over time [*F*_(3, 33)_ = 10.94, *p* < 0.005]. No interaction effect was observed [*F*_(3, 33)_ = 1.77, *p* > 0.05]. The increased power at gamma frequency in CFA inflammatory pain rats occurred at day 28 after CFA injection (*p* < 0.05, Bonferroni *post hoc* test). These results indicated that gamma activity from the whole brain areas was enhanced in CFA rats.

Furthermore, we calculated the spatial distribution of the gamma power over the electrodes to figure out the areas where the enhanced gamma activities located. The topography of gamma-band power over the electrodes in two groups at each time point was calculated and plotted. As shown in Figure 2C, compared with the NS group, the CFA group showed increased gamma power in channels 3 and 10 at day 28 [*F*_(1, 11)_ = 5.05, *p* < 0.05; *p* < 0.05, Bonferroni *post hoc* test]. The main effect of time was significant [*F*_(3, 33)_ = 15.66, *p* < 0.0001], indicating that gamma power increased gradually during the development of chronic inflammatory pain. No interaction effect was observed [*F*_(3, 33)_ = 0.97, *p* > 0.05].

### Gamma activity correlated with hyperalgesia

To explore the potential function of enhanced gamma activity in chronic pain and to test whether gamma activity participates in hyperalgesia, gamma power averaged across recording channels was further correlated with hyperalgesia measured with the laser energy that elicited pain response. Data at D7, D14, and D28 from chronic pain rats were collected. A significant negative correlation between gamma power and the laser energy was observed (Pearson γ = −0.45, *p* < 0.05; Figure 2D), indicating a correlation of stronger gamma power with lower laser energy, i.e., stronger gamma power, higher hyperalgesia.

### Enhanced coupling between the phase of theta and the amplitude of gamma in chronic inflammatory pain rats

**Figure 3:**
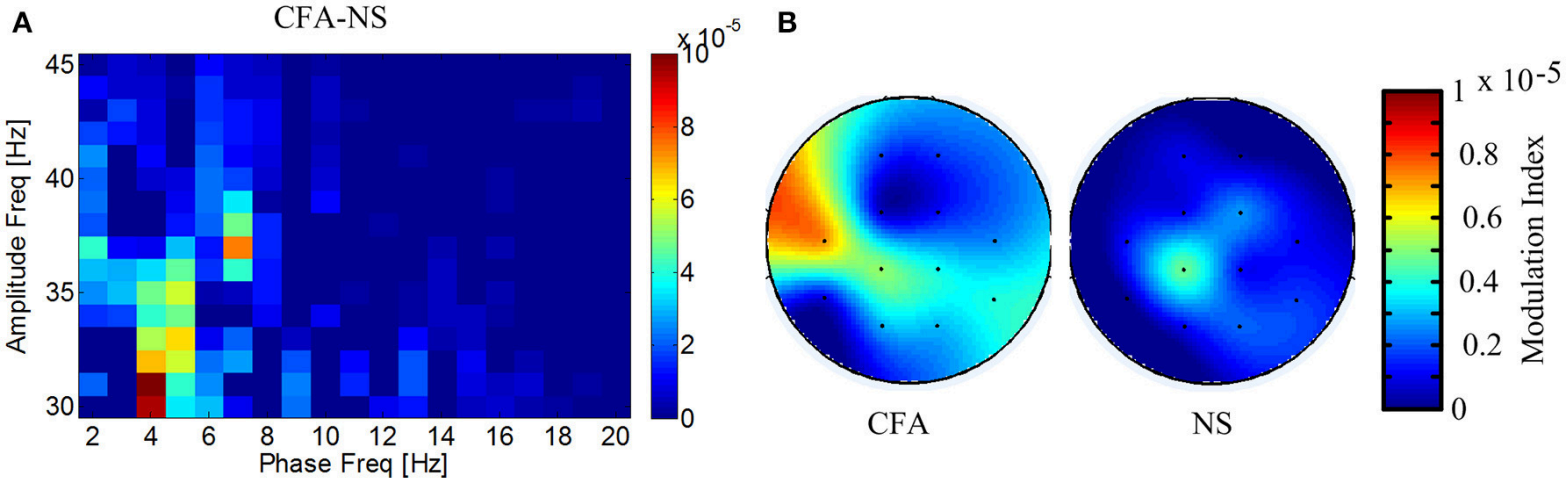
**Enhanced coupling between gamma amplitude and theta phase. (A)** Difference of phase-amplitude coupling between chronic pain and control. **(B)** Gamma power had strong modulation index with theta phase in CFA chronic pain rats.

Since gamma power is coupling with theta phase in acute pain (Wang et al., 2011) and it is usually modulated by the phase of theta oscillation, we further tested whether theta phase modulates gamma activity in chronic inflammatory pain. Coupling between the amplitude of gamma and the phase of low frequency (2–20 Hz) was calculated for each channel for both groups at day 28, and the difference value of cross-frequency coupling index averaged across channels between chronic pain and control was plotted. Figure 3A showed the gamma amplitude was coupling with the phase of oscillations mainly at the theta frequency band (4–8 Hz). Then, we compared the theta-gamma phase amplitude coupling between chronic pain and control condition. Results showed that the phase-amplitude coupling between theta band and gamma band was significantly different between two groups [*F*_(1, 11)_ = 4.02, *p* < 0.05; Figure 3B], indicating that the theta phase significantly modulated the gamma power in chronic inflammatory pain.

## Discussion

In the present study, we found a significant increase of gamma oscillatory activity in rats with chronic inflammatory pain. The enhanced gamma activity correlated with the hyperalgesia and had a coupling with the phase of theta oscillation.

### Enhanced gamma activity in rats with chronic inflammatory pain

Although there are several studies on gamma oscillations in experimental brief and tonic pain in healthy human beings and in normal rats (Croft et al., 2002; Gross et al., 2007; Wang et al., 2011; Hu et al., 2014; Liu et al., 2015b,a; Schulz et al., 2015; Li et al., 2016), as well as convert pain in patients with chronic disorders of consciousness (Naro et al., 2016), knowledge about the spontaneous and task-free gamma oscillatory activity in pathological, long-lasting chronic pain state are still in expectation. Our present study found that ECoG gamma activity increased under chronic inflammatory pain condition in a CFA monoarthritis model of rats. This CFA model mimics the whole processes from acute stage to chronic stage of inflammatory pain (Butler et al., 1992). Results showed that gamma power gradually reached a significantly high level at the chronic pain states. These findings suggested that the enhanced gamma oscillatory activity was related to long-lasting pain condition, and extended our understanding of gamma activity from physiological pain to pathological pain conditions.

We found that gamma activity under chronic inflammatory pain condition enhanced over two electrodes over bilateral brain regions. The location of these electrodes was at 3.0 mm posterior and ±4.5 mm lateral to the bregma. According to the atlas of rat brain (George Paxinos, 2004), these areas are the location of primary somatosensory cortices. Primary somatosensory cortex encodes the sensory component of pain. This is in line with previous studies that brief painful stimuli evoked gamma activity in the primary somatosensory cortex of human beings (Gross et al., 2007). The gamma activity induced by brief noxious stimulus was observed in the contralateral somatosensory cortex as measured by magnetoencephalography (MEG) and local filed potentials from human beings. Unlike gamma activity induced by brief pain, the gamma activity in our chronic pain model increased in bilateral brain. Because the channel location of ECoG is less accurate than the source location of MEG and the local field potentials, increased activity in bilateral brain areas cannot rule out the contribution from activities of other brain regions which exhibited bilateral activation during chronic pain (Hashmi et al., 2013).

Increased gamma activity may result from the increased GABAergic activity. It is known that neurotransmitter GABA is critical for the generation of gamma oscillation and thus the gamma activity depends on GABA activity (Barr et al., 2013). A recent study showed increased GABA concentrations in inflammatory pain rats (Amirmohseni et al., 2016). So, the increased gamma activity in chronic inflammatory pain was consistent with the increased GABA concentration.

### Correlation of gamma oscillation with hyperalgesia

Correlation analysis showed that gamma power was positively correlated to hyperalgesia of chronic pain (Figure 3). Previous studies demonstrated that gamma oscillatory activity encoded acute or tonic subjective pain intensity on human beings (Gross et al., 2007; Wang et al., 2011; Peng et al., 2014; Tiemann et al., 2015), our current study on CFA pain rats extended the explanation of this correlation of gamma power with pain intensity under chronic inflammatory pain condition. First, the measurement of pain intensity was different. In the studies on brief or tonic pain, pain intensity was evaluated by subjective rating induced by painful stimulus. It reflected the intensity of pain perception to painful stimulus. In our chronic pain conditions, pain intensity was measured by the laser energy that could elicit pain response. It reflected the pain sensitivity or the intensity of hyperalgesia and allodynia of rats but not perception of a single stimulus. Second, the EEG recording and analysis were different. In brief or acute pain conditions, EEG was recorded with external stimulation to estimate stimulus-induced gamma activity. In our present study, ongoing EEG was used to estimate the brain activities in resting state in chronic pain conditions. Therefore, our observations likely indicated that the activity of neural gamma oscillations at resting state correlated to hyperalgesia and severity of chronic pain. Indeed, a recent research which showed that the pre-stimulus spontaneous gamma oscillations predict the subsequent pain perception was consistent with our findings (Tu et al., 2016).

It should be noted that except the brain activities in resting state, ongoing EEG signals may contain activities related to inflammatory ongoing pain. However, a recent research reported that the ongoing pain was transient in rats with chronic inflammatory pain, appearing at 24 h after inflammation induction but disappearing at 4 days (Okun et al., 2011). Accordingly, our ECoG signals from D7, D14, and D28 hardly reflect the ongoing pain, and it was also the reason that we did not measure the ongoing pain behavior and correlated with gamma oscillation.

### Cross-frequency coupling between gamma amplitude and theta phase

It is known that gamma frequency activities generate from the recurrent inhibitory–excitatory neural network. In the cortical network, this local gamma network receives inputs from thalamic inputs and other brain areas. These inputs exhibits phase variations in the low frequency range (Mazzoni et al., 2010, 2011). Cross frequency coupling between phase of low frequency and gamma power reflects the modulation of sensory and other inputs to local neural networks (Mazzoni et al., 2010, 2011). Thus, the enhanced cross-frequency coupling in chronic pain suggested that modulation from thalamic and other areas to primary sensory cortices is increased in chronic pain. This is supported by clinic findings that the theta frequency from the thalamic nuclei is increased in chronic pain (Stern et al., 2006) and is consistent with idea that connectivity of brain network serving in pain is increased in chronic pain conditions (Baliki et al., 2014).

## Limitations

Our study is interesting but preliminary. There are several factors need to be considered. First, the rat model of chronic pain cannot fully represent chronic pain patients. However, ECoG of rats could prevent the contamination of muscle activity which is usually accompanied by pain and allow chronic recordings along the development of chronic pain. Second, ECoGs were recorded at resting state for several minutes to reflect the brain state under chronic pain condition. But, it is unavailable for accurate estimation of the function of gamma activities in the present study, because there is no specific event-related information in the ongoing EEG signals. Third, due to limited channels of rat ECoG, source location of gamma activity and cross-frequency coupling are hard to be estimated. Besides, small sample size is a weak point because of long time recordings. Nevertheless, our results provide first evidence for the enhanced gamma activity in chronic pain conditions even without external painful stimulation.

Our results demonstrate that the gamma power is enhanced in chronic inflammatory pain and correlated with hyperalgesia. It extends our understanding about gamma activity in pain that gamma power serves as a possible biomarker of severity of chronic pain.

## Author contributions

JW (the first author) carried out the ECoG recording and data analysis, participated in the study design and drafted the manuscript. JW (the second author) carried out the ECoG recording and the preprocessing of data. GX participated in the design of the study. XL carried out the data analysis and helped to draft the manuscript. YW conceived of the study, and participated in its design and helped to draft the manuscript. All authors read and approved the final manuscript.

### Conflict of interest statement

The authors declare that the research was conducted in the absence of any commercial or financial relationships that could be construed as a potential conflict of interest.
